# Terminating Basic Hypergeometric Representations and Transformations for the Askey–Wilson Polynomials

**DOI:** 10.3390/sym12081290

**Published:** 2020

**Authors:** Howard S. Cohl, Roberto S. Costas-Santos, Linus Ge

**Affiliations:** 1 Applied and Computational Mathematics Division, National Institute of Standards and Technology, Mission Viejo, CA 92694, USA; 2 Departamento de Física y Matemáticas, Universidad de Alcalá, 28871 Alcalá de Henares, Spain; 3 Department of Mathematics, University of Rochester, Rochester, NY 14627, USA

**Keywords:** basic hypergeometric series, basic hypergeometric orthogonal polynomials, basic hypergeometric transformations

## Abstract

In this survey paper, we exhaustively explore the terminating basic hypergeometric representations of the Askey–Wilson polynomials and the corresponding terminating basic hypergeometric transformations that these polynomials satisfy.

## 1. Introduction

This paper is a study in *q*-calculus (typically taken with |*q*| *<* 1). The *q*-calculus (introduced by such luminaries as Leonhard Euler, Eduard Heine and Garl Gustav Jacobi) is a calculus of finite differences which becomes the standard infinitesimal calculus (introduced by Isaac Newton and Gottfried Wilhelm Leibniz) in the limit as *q* → 1. The work contained in this paper is directly connected to properties of the Askey–Wilson polynomials *p*_*n*_(*x*; **a**|*q*) ([1] §14.1) which are at the very top of the *q*-Askey scheme (see e.g., ([1] Chapter 14)). The Askey–Wilson polynomials are basic hypergeometric orthogonal polynomials with interpretations in quantum group theory, combinatorics, and probability. The applications of Askey–Wilson polynomials include invariants of links, *3*-manifolds and *6j*-symbols (see e.g., [2]). The definition of the Askey–Wilson polynomials in terms of terminating basic hypergeometric series are given in Theorem 3 below. The Askey–Wilson polynomials are symmetric with respect to their four free parameters, that is, they remain unchanged upon interchange of any two of the four free parameters. It should be emphasized that since 1970, the subjects of special functions and special families of orthogonal polynomials have gone through major developments, of which the study of the Askey–Wilson polynomials has been central. Many of the properties of these polynomials can be derived from their terminating basic hypergeometric representations, so an exhaustive catalog of these representations, as contained here, will be quite convenient for lookup.

The Askey–Wilson polynomials can be defined in terms of terminating basic hypergeometric series (see, e.g., (11)), which in turn are defined in terms of a sum of products of *q*-Pochhammer symbols. Using the properties of *q*-Pochhammer symbols, it is straightforward to replace q↦1/q in the complex plane in order to obtain an extension of these polynomials with |*q*| *>* 1. One often refers to these polynomials obtained as *q*^−1^ or 1/*q* polynomials. Since these algebraic factors are difficult to search on in the literature, we refer to this extension specifically as the *q*-inverse polynomials.

It should however be noted that while the Askey–Wilson polynomials represent an infinite-family of orthogonal polynomials $\left(n\in {\mathbb{N}}_{0}\right)$, orthogonal with respect to a weight function on [−1, 1] ([1] (14.1.2)) (which gives restrictions on the values of the free parameters), the *q*-inverse Askey–Wilson polynomials represent a finite-family of basic hypergeometric orthogonal polynomials $\left(n\in \left\{0,\ldots ,N\right\},N\in {\mathbb{N}}_{0}\right)$ (see e.g., [3]). Not only that, but it is also known that the *q*-inverse Askey–Wilson polynomials are simply a scaled version of the Askey–Wilson polynomials with their parameters replaced by their reciprocals (see Remark 8, below).

In the sequel, from the basic terminating hypergeometric representations of the Askey–Wilson polynomials, we derive terminating basic hypergeometric representations for the *q*-inverse Askey–Wilson polynomials (the Askey–Wilson polynomials with *q* replaced by 1/*q*). From the terminating basic hypergeometric representations of the Askey–Wilson polynomials, one can easily derive transformation formulae for terminating basic hypergeometric functions.

The main focus of this survey paper will be to exhaustively describe the transformation identities for the terminating basic hypergeometric functions which appear as representations for these polynomials. Some of these transformation identities are well-known in the literature, but we also give the transformation identities for these basic hypergeometric functions which are obtained by the symmetry of the polynomials under parameter interchange, and under the map θ↦−θ, for *x* = cos *θ*. It should be noted that the symmetry group *G* of these functions coincide with the symmetry group of the very-well poised _8_*W*_7_ which is a subgroup *WD*_5_ of *WB*_5_, the Weyl group of a root system of type *B*_*n*_. The order of *G* is 5!2^4^ = 1920. For more details, see [4–6].

## 2. Preliminaries

We adopt the following set notations: ${\mathbb{N}}_{0}:=\left\{0\right\}\cup \mathbb{N}=\left\{0,1,2,\ldots \right\}$, and we use the sets ℤ, ℝ, ℂ which represent the integers, real numbers and complex numbers respectively, ${\mathbb{C}}^{*}:=\mathbb{C}\backslash \left\{0\right\}$. We also adopt the following notation and conventions. Let **a** := {*a*_1_, *a*_2_, *a*_3_, *a*_4_}, *b*, $a_{k}\in \mathbb{C}$, *k* = 1, 2, 3, 4. Define **a** + *b* := {*a*_1_ + *b*, *a*_2_ + *b*, *a*_3_ + *b*, *a*_3_ + *b*}, *a*_12_ := *a*_1_*a*_2_, *a*_13_ := *a*_1_*a*_3_, *a*_23_ := *a*_2_*a*_3_, *a*_123_ := *a*_1_*a*_2_*a*_3_, *a*_1234_ := *a*_1_*a*_2_*a*_3_*a*_4_, etc. Throughout the paper, we assume that the empty sum vanishes and the empty product is unity.

**Definition 1.** Throughout this paper we adopt the following conventions for succinctly writing elements of lists. To indicate sequential positive and negative elements, we write

±a:={a,−a}.

We also adopt an analogous notation

$$e^{\pm i\theta }:=\left\{e^{i\theta },e^{-i\theta }\right\}.$$

In the same vein, consider a finite sequence $f_{s}\in \mathbb{C}$ with s∈S⊂ℕ. Then, the notation {*f*_*s*_} represents the sequence of all complex numbers *f*_*s*_ such that s∈S. Furthermore, consider some p∈S, then the notation ${\left\{f_{s}\right\}}_{s\neq p}$ represents the sequence of all complex numbers *f*_*s*_ such that s∈S\{p}. In addition, for the empty list, *n* = 0, we take

$$\left\{a_{1},\ldots ,a_{n}\right\}:=Ø.$$

Consider $q\in {\mathbb{C}}^{*}$ such that |q|≠1. Define the sets $\Omega_{q}^{n}:=\left\{q^{-k}:n,k\in {\mathbb{N}}_{0},0\leq k\leq n-1\right\}$, $\Omega_{q}:=\Omega_{q}^{\infty }=\left\{q^{-k}:k\in {\mathbb{N}}_{0}\right\}$. In order to obtain our derived identities, we rely on properties of the *q*-Pochhammer symbol (*q*-shifted factorial). For any $n\in {\mathbb{N}}_{0}$, *a*, q∈ℂ, the *q*-Pochhammer symbol is defined as

(1) $${\left(a;q\right)}_{n}:=\left(1-a\right)\left(1-aq\right)\cdots \left(1-aq^{n-1}\right),\;n\in {\mathbb{N}}_{0}.$$

One may also define

(2) $${\left(a;q\right)}_{\infty }:=\prod_{n=0}^{\infty }\left(1-aq^{n}\right),$$

where |*q*| *<* 1. Furthermore, define

$${\left(a;q\right)}_{b}:=\frac{{\left(a;q\right)}_{\infty }}{{\left(aq^{b};q\right)}_{\infty }}.$$

where $aq^{b}\notin \Omega_{q}$. We will also use the common notational product conventions

$${\left(a_{1},\ldots ,a_{k}\right)}_{b}:={\left(a_{1}\right)}_{b}\cdots {\left(a_{k}\right)}_{b},$$

$${\left(a_{1},\ldots ,a_{k};q\right)}_{b}:={\left(a_{1};q\right)}_{b}\cdots {\left(a_{k};q\right)}_{b}.$$

The following properties for the *q*-Pochhammer symbol can be found in Koekoek et al. ([1] (1.8.7), (1.8.10–11), (1.8.14), (1.8.19), (1.8.21–22)), namely for appropriate values of *q*, $a\in {\mathbb{C}}^{*}$ and *n*, $k\in {\mathbb{N}}_{0}$:

(3) $${\left(a;q^{-1}\right)}_{n}={\left(a^{-1};q\right)}_{n}{\left(-a\right)}^{n}q^{-{(}_{2}^{n})},$$

(4) $${\left(a;q\right)}_{n+k}={\left(a;q\right)}_{k}{\left(aq^{k};q\right)}_{n}={\left(a;q\right)}_{n}{\left(aq^{n};q\right)}_{k},$$

(5) $${\left(a;q\right)}_{n}={\left(q^{1-n}/a;q\right)}_{n}{\left(-a\right)}^{n}q^{{(}_{2}^{n})},$$

(6) $${\left(aq^{-n};q\right)}_{k}=q^{-nk}\frac{{\left(q/a;q\right)}_{n}}{{\left(q^{1-k}/a;q\right)}_{n}}{\left(a;q\right)}_{k},$$

(7) $${\left(a^{2};q^{2}\right)}_{n}={\left(\pm a;q\right)}_{n},$$

(8) $${\left(a;q\right)}_{2n}={\left(a,aq,q^{2}\right)}_{n}={\left(\pm \sqrt{a},\pm \sqrt{qa};q\right)}_{n}.$$

Observe that by using (1) and (7), one obtains

(9) $${\left(aq^{n};q\right)}_{n}=\frac{{\left(\pm \sqrt{a},\pm \sqrt{aq};q\right)}_{n}}{{\left(a;q\right)}_{n}},\;a\notin \Omega_{q}^{n}.$$

The basic hypergeometric series, which we will often use, is defined for q, $z\in {\mathbb{C}}^{*}$ such that |*q*|, |*z*| *<* 1, *s*, $r\in {\mathbb{N}}_{0}$, $b_{j}\notin \Omega_{q}$, *j* = 1, …, *s*, as ([1] (1.10.1))

(10) ϕr s(a1,…,arb1,…,bs;q,z):=∑k=0∞(a1,…,ar;q)k(q,b1,…,bs;q)k((−1)kq( 2k))1+s−rzk.

Note that we refer to a basic hypergeometric series as *ℓ*-balanced if $q^{\ell }a_{1}\cdots a_{r}=b_{1}\cdots b_{s}$, and balanced if ℓ=1. A basic hypergeometric series ϕr+1 r is well-poised if the parameters satisfy the relations

$$qa_{1}=b_{1}a_{2}=b_{2}a_{3}=\cdots =b_{r}a_{r+1}.$$

It is very-well poised if in addition, $\left\{a_{2},a_{3}\right\}=\pm q\sqrt{a_{1}}.$

Similarly for terminating basic hypergeometric series which appear in basic hypergeometric orthogonal polynomials, one has

(11) ϕr s(q−n,a1,…,ar−1b1,…,bs;q,z):=∑k=0n(q−n,a1,…,ar−1;q)k(q,b1,…,bs;q)k((−1)kq( 2k))1+s−rzk,

where $b_{j}\notin \Omega_{q}^{n}$, j=1,…,s. Define the very-well poised basic hypergeometric series _*r*+1_*W*_*r*_ ([7] (2.1.11))

(12) Wr+1 r(b;a4,…,ar+1;q,z):=ϕr+1 r(b,±qb,a4,…,ar+1±b,qba4,…,qbar+1;q,z),

where $\sqrt{b},\frac{qb}{a_{4}},\ldots ,\frac{qb}{a_{r+1}}\notin \Omega_{q}$. When the very-well poised basic hypergeometric series is terminating, then one has

(13) Wr+1 r(b;q−n,a5,…,ar+1;q,z):=ϕr+1 r(b,±qb,q−n,a5,…,ar+1±b,qn+1b,qba5,…,qbar+1;q,z),

where $\sqrt{b},\frac{qb}{a_{5}},\ldots ,\frac{qb}{a_{r+1}}\notin \Omega_{q}^{n}\cup \left\{0\right\}$. The Askey–Wilson polynomials are intimately connected with the terminating very-well poised _8_*W*_7_, which is given by

(14)  8W7(b;q−n,c,d,e,f,q,z):=ϕ8 7(b,±qb,q−n,c,d,e,f±b,qn+1b,qbc,qbd,qbe,qbf;q,z),

where $\sqrt{b},\frac{qb}{c},\frac{qb}{d},\frac{qb}{e},\frac{qb}{f}\notin \Omega_{q}^{n}\cup \left\{0\right\}$.

Some classical transformations for basic hypergeometric series which we will use include Watson’s *q*-analog of Whipple’s theorem which relates a terminating balanced _4_*ϕ*_3_ to a terminating very-well poised _8_*W*_7_ (cf. ([8] (17.9.15)))

(15) ϕ4 3(q−n,a,b,cd,e,f;q,q)=(deab,deac;q)(dea,deabc;q) 8W7(deqa;q−n,da,ea,b,c;q,qaf),

where *qabc* = *de f*.

In ([7] Exercise 1.4ii), one finds the inversion formula for terminating basic hypergeometric series.

**Theorem 1** (Gasper and Rahman (1990)). Let *m*, *n*, *k*, *r*, $s\in {\mathbb{N}}_{0}$, 0 ≤ *k* ≤ *r,* 0 ≤ *m* ≤ *s*, $a_{k}\in \mathbb{C}$, $b_{m}\notin \Omega_{q}^{n}$, $q\in {\mathbb{C}}^{*}$ such that |q|≠1. Then,

(16) ϕr+1 s(q−n,a1,…,arb1,…,bs;q,z)=(a1,…,ar;q)n(b1,…,bs;q)n(zq)n((−1)nq(2n))s−r−1        ×∑k=0n(q−n,q1−nb1,…,q1−nbs;q)k(q,q1−na1,…,q1−nar;q)k(b1⋯bsa1⋯arqn+1z)k.

**Corollary 1.** Let *n*, $r\in {\mathbb{N}}_{0}$, $q\in {\mathbb{C}}^{*}$ such that |q|≠1, and for 0 ≤ *k* ≤ *r*, let *a*_*k*_, $b_{k}\notin \Omega_{q}^{n}\cup \left\{0\right\}$. Then,

(17) ϕr+1 r(q−n,a1,…,arb1,…,br;q,z)=(−1)nq−(2n)(a1,…,ar;q)n(b1,…,br;q)nϕr+1 r(q−n,q1−nb1,…,q1−nbrq1−na1,…,q1−nar;q,qn+1zb1⋯bra1⋯ar).

**Proof.** Take *r* = *s*, in (16) and using the definition (10) completes the proof. □

Note that in Corollary 1 if the terminating basic hypergeometric series on the left-hand side is balanced then the argument of the terminating basic hypergeometric series on the right-hand side is *q*^2^/*z*.

Applying Corollary 1 to the definition of _*r*+1_*W*_*r*_, we obtain the following result for a terminating very-well poised basic hypergeometric series _*r*+1_*W*_*r*_.

**Corollary 2.** Let $n\in {\mathbb{N}}_{0}$, *b*, *a*_*k*_, *q*, $z\in {\mathbb{C}}^{*}$, $\sqrt{b}$, $q^{n+1}b$, $\frac{qb}{a_{k}}$, $\frac{q^{1-n}}{b}$, $\frac{q^{1-n}}{a_{k}}\notin \Omega_{q}^{n}$, *k* = 5, …, *r* + 1. Then one has the following transformation formula for a very-well poised terminating basic hypergeometric series:

Wr+1 r(b;q−1,a5,…,ar+1;q,z)=q−(2n)(−zq)n(±qb,b,a5,…,ar+1;q)n(±qb,qn+1b,qba5,…,qbar+1;q)n         ×Wr+1 r(q−2nb;q−n,q−na5b,⋯,q−nar+1b;q,q2n+r−3br−3(a5⋯ar−1)2z).

**Proof.** Use Corollary 1 and (13). □

An interesting and useful consequence of this formula is the *r* = 7 special case,

(18) $$\begin{array}{l} {\;}_{8}W_{7}\left(b;q^{-n},c,d,e,f;q,z\right)=q^{-{(}_{2}^{n})}{\left(\frac{-z}{q}\right)}^{n}\frac{{\left(\pm q\sqrt{b},b,c,d,e,f;q\right)}_{n}}{{\left(\pm q\sqrt{b},q^{n+1}b,\frac{qb}{c},\frac{qb}{d},\frac{qb}{e},\frac{qb}{f};q\right)}_{n}}\\ \;\;\;\;\;\;\;\;\;\times {\;}_{8}W_{7}\left(\frac{q^{-2n}}{b};q^{-n},\frac{q^{-n}c}{b},\frac{q^{-n}d}{b},\frac{q^{-n}e}{b},\frac{q^{-n}f}{b};q,\frac{q^{2n+4}b^{4}}{z{\left(cdef\right)}^{2}}\right). \end{array}$$

Note that in the case when the one obtains an _8_*W*_7_ from a balanced _4_*ϕ*_3_ using (15), then *q*^2*n*+4^*b*^4^/(*z*(*cde f*)^2^) = *z*.

Another equality we can use is the following connecting relation between basic hypergeometric series on *q*, and on *q*^−1^:

(19) ϕr+1 r(q−n,a1,…,arb1,…,br;q,z)=ϕr+1 r(qn,a1−1,…,ar−1b1−1,…,br−1;q−1.a1a2⋯arb1b2⋯brzqn+1)    =(a1,…,ar;q)n(b1,…,br;q)n(−zq)nq−(2n)ϕr+1 r(q−n,q1−nb1,…,q1−nbrq1−na1,…,q1−nar;q,b1⋯bra1⋯arqn+1z)

In order to understand the procedure for obtaining the *q*-inverse analogues of the basic hypergeometric orthogonal polynomials studied in this manuscript, let’s consider a special case in detail. Let *f*_*r*_(*q*) := *f*_*r*_(*q*; *z*(*q*); **a**(*q*), **b**(*q*)) be defined as

(20) fr(q):=gr(q)ϕr+1 r(q−n,a(q)b(q);q,z(q)),

where

$$\begin{array}{c} a\left(q\right):=\left\{a_{1}\left(q\right),\ldots ,a_{r}\left(q\right)\right\}\\ b\left(q\right):=\left\{b_{1}\left(q\right),\ldots ,b_{r}\left(q\right)\right\} \end{array}\},$$

which will suffice for instance, for the study of the terminating basic hypergeometric representations for the Askey–Wilson polynomials. In order to obtain the corresponding *q*-inverse hypergeometric representations of *f*_*r*_(*q*), one only needs to consider the corresponding *q*-inverted function:

(21) fr(q−1):=gr(q−1)ϕr+1 r(qn,a(q−1)b(q−1);q−1,z(q−1)).

**Theorem 2.** Let *r*, $k\in {\mathbb{N}}_{0}$, 0 ≤ *k* ≤ *r*, $a_{k}\left(q\right)\in \mathbb{C}$, $b_{k}\left(q\right)\notin \Omega_{q}$, $q\in {\mathbb{C}}^{*}$ such that |q|≠1, z(q)∈ℂ Define a multiplier function *g*_*r*_(*q*) := *g*_*r*_(*q*; *z*(*q*); **a**(*q*); **b**(*q*)) which is not of basic hypergeometric type (some multiplicative combination of powers and q-Pochhammer symbols), and *z*(*q*) := *z*(*q*; **a**(*q*); **b**(*q*)). Then, define *f*_*r*_(*q*) as in (20), and one has

(22) fr(q−1)=gr(q−1)ϕr+1 r(qn,a−1(q−1)b−1(q−1);q,qn+1a1(q−1)⋯ar(q−1)z(q−1)b1(q−1)⋯br(q−1)),

where

$$\begin{array}{c} a^{-1}\left(q^{-1}\right)=\left\{\frac{1}{a_{1}\left(q^{-1}\right)},\ldots ,\frac{1}{a_{r}\left(q^{-1}\right)}\right\}\\ b^{-1}\left(q^{-1}\right)=\left\{\frac{1}{b_{1}\left(q^{-1}\right)},\ldots ,\frac{1}{b_{r}\left(q^{-1}\right)}\right\} \end{array}\}.$$

**Proof.** Using the identity (3) repeatedly with the definition (10), in (21), obtains the *q*-inverted terminating representation (22) which corresponds to the original terminating basic hypergeometric representation (20). This completes the proof. □

We will obtain new transformations for basic hypergeometric orthogonal polynomials by taking advantage of the following remark.

**Remark 1.** Since *x* = cos *θ* is an even function of θ, all polynomials in cos *θ* will be invariant under the map θ↦−θ.

**Remark 2.** Observe in the following discussion we will often be referring to a collection of constants *a*, *b*, *c*, *d*, *e*, *f*. In such cases, which will be clear from context, then the constant e should not be confused with Euler’s number, the base of the natural logarithm, i.e., log e = 1.

## 3. The Askey–Wilson Polynomials

Define the sets **4** := {1, 2, 3, 4}, **a** := (*a*_1_, *a*_2_, *a*_3_, *a*_4_), $a_{k}\in {\mathbb{C}}^{*}$, *k* ∈ **4**, and *x* = cos *θ* ∈ [−1, 1]. The Askey–Wilson polynomials *p*_*n*_(*x*; **a**|*q*) are a family of polynomials symmetric in four free parameters. These polynomials have a long and in-depth history and their properties have been studied in detail. The basic hypergeometric series representation of the Askey–Wilson polynomials fall into four main categories: (1) terminating _4_*ϕ*_3_ representations; (2) terminating _8_*W*_7_ representations; (3) nonterminating _8_*W*_7_ representations; and (4) nonterminating _4_*ϕ*_3_ representations.

One may obtain alternative nonterminating representations of the Askey–Wilson polynomials using ([7] (2.10.7)), namely

(23) ϕ4 3(q−n,qn−1a1234,ape±iθ{aps}s≠p;q,q)=(q1−ne2iθ,q1−natu,q2−neiθaprt,q2−neiθapru;q)∞(q1−neiθat,q1−neiθau,q2−ne2iθapr,q2−na1234;q)∞        × 8W7(q1−ne2iθapr,q1−napr,qeiθap,qeiθar,ateiθ,aueiθ;q,q1−natu),

provided |*q*^1−*n*^/*a*_*tu*_| *<* 1. However, there exist no fixed values of |*a*_*k*_|, |*q*| *<* 1 such that this very-well-poised _8_*W*_7_ is convergent for all $n\in {\mathbb{N}}_{0}$. On the other hand, it is possible to find nonterminating _8_*W*_7_ representations which are convergent for all $n\in {\mathbb{N}}_{0}$. It is also possible to find nonterminating _4_*ϕ*_3_ representations of the Askey–Wilson polynomials using Bailey’s transformation of a very-well-poised _8_*W*_7_ ([8] (17.9.16))

(24)  8W7(b;a,c,d,e,f;q,q2b2acdef)=(qb,qbde,qbdf,qbef;q)∞(qbd,qbe,qbf,qbdef;q)∞ϕ4 3(qbac,d,e,fqba,qbc,defb;q,q)       +(qb,qbac,d,e,f,q2b2adef,q2b2cdef;q)∞(qba,qbc,qbd,qbe,qbf,q2b2acdef,defqb;q)∞ϕ4 3(qbde,qbdf,qbef,q2b2acdefq2b2adef,q2b2cdef,q2bdef;q.q).

However, these nonterminating representations will not be further discussed in this paper.

Different series representations are useful for obtaining different properties and formulae for these polynomials. So it is very useful to have at hand an exhaustive list. The discussion contained in this section is an attempt to summarize, in an in-depth manner, an exhaustive description of the representation and transformation properties of the terminating _4_*ϕ*_3_ and _8_*W*_7_ basic hypergeometric representations of the Askey–Wilson polynomials.

### 3.1. The Askey–Wilson Polynomial Representations

**Theorem 3.** Let $n\in {\mathbb{N}}_{0}$, *p*, *s*, *r*, *t*, *u* ∈ **4**, *p*, *r*, *t*, *u* distinct and fixed, $q\in {\mathbb{C}}^{*}$ such that |q|≠1. Then, the Askey–Wilson polynomials have the following terminating basic hypergeometric series representations given by:

(25) pn(x;a|q):=ap−n({aps}s≠p;q)nϕ4 3(q−n,qn−1a1234,ape±iθ{aps}s≠p;q,q)

(26) =q−(2n)(−ap)−n(a1234q;q)2n(ape±iθ;q)n(a1234q;q)nϕ4 3(q−n,{q1−naps}s≠pq2−2na1234,q1−ne±iθap;q,q)

(27) =einθ(apr,ate−iθ,aue−iθ;q)nϕ4 3(q−n,apeiθ,areiθ,q1−natuapr,q1−neiθat,q1−neiθau;q,q)

(28) $$=e^{in\theta }\frac{{\left(\frac{a_{1234}}{q};q\right)}_{2n}{\left({\left\{a_{s}e^{-i\theta }\right\}}_{s\neq p},\frac{a_{1234}e^{-i\theta }}{qa_{p}};q\right)}_{n}}{{\left(\frac{a_{1234}}{q};q\right)}_{n}{\left(\frac{a_{1234}e^{-i\theta }}{qa_{p}};q\right)}_{2n}}\;\;\;\;\;\;\;\times {\;}_{8}W_{7}\left(\frac{q^{1-2n}a_{p}e^{i\theta }}{a_{1234}};q^{-n},{\left\{\frac{q^{1-n}a_{ps}}{a_{1234}}\right\}}_{s\neq p},a_{p}e^{i\theta };q,\frac{qe^{i\theta }}{a_{p}}\right)$$

(29) $$=e^{in\theta }\frac{{\left(a_{p}e^{-i\theta },{\left\{\frac{a_{1234}}{a_{ps}}\right\}}_{s\neq p};q\right)}_{n}}{{\left(\frac{a_{1234}e^{i\theta }}{a_{p}};q\right)}_{n}}{\;}_{8}W_{7}\left(\frac{a_{1234}e^{i\theta }}{qa_{p}};q^{-n},{\left\{a_{s}e^{i\theta }\right\}}_{s\neq p},q^{n-1}a_{1234};q,\frac{qe^{-i\theta }}{a_{p}}\right)$$

(30) $$=a_{p}^{-n}\frac{{\left(a_{pt},a_{pu},a_{r}e^{\pm i\theta };q\right)}_{n}}{{\left(\frac{a_{r}}{a_{p}};q\right)}_{n}}{\;}_{8}W_{7}\left(\frac{q^{-n}a_{p}}{a_{r}};q^{-n},\frac{q^{1-n}}{a_{rt}},\frac{q^{1-n}}{a_{ru}},a_{p}e^{\pm i\theta };q,q^{n}a_{tu}\right)$$

(31) $$=e^{in\theta }\frac{{\left(\left\{a_{s}e^{-i\theta }\right\};q\right)}_{n}}{{\left(e^{-2i\theta };q\right)}_{n}}{\;}_{8}W_{7}\left(q^{-n}e^{2i\theta };q^{-n},\left\{a_{s}e^{i\theta }\right\};q,\frac{q^{2-n}}{a_{1234}}\right).$$

**Proof.** The standard definition of the Askey–Wilson polynomials (25) is found in many places including ([1] (14.1.1)). The representation (26) can be derived by applying (16) to (25). It also follows by using ([8] second equality in (17.9.14)) with *a* = *a*_1234_*q*^*n*−1^, {*b*, *c*} = *a*_*p*_e^±*iθ*^, $\left\{d,e,f\right\}={\left\{a_{ps}\right\}}_{s\neq p}$. One can obtain (27) by starting with (25) and *p* ↔ *u* using ([8] second equality in (17.9.14)) with $\left\{a,b\right\}=a_{u}e^{\mp i\theta }$, *c* = *a*_1234_*q*^*n*−1^, $\left\{d,e,f\right\}={\left\{a_{us}\right\}}_{s\neq u}$, and ([1] (1.8.14)). The representation (28) follows by from (29) by using the inversion formula (18). The representation (29) follows from (25) with *p* ↔ *r*, using Watson’s *q*-analogue of Whipple’s theorem (15) and mapping θ↦−θ. The representation (30) follows by using ([7] (III.19)) directly with (25). The representation (31) follows from (25) and ([7] (III.15), (III.19)) (see also ([9] §14.1)). This completes the proof. □

Note that when Corollary 1 is applied to (25) one obtains (26), and when one applies it to (27), one obtains the same formula back with θ↦−θ and {*r*, *s*} ↔ {*t*, *u*}.

**Remark 3.** Applying (16) to (27), (28), (29), (31) simply takes θ↦−θ, and applying it to (30) interchanges *a*_*p*_ and *a*_*r*_. Mapping θ↦−θ may give additional representations, however those are omitted.

### 3.2. Terminating 4-Parameter Symmetric Transformations

**Corollary 3.** Let $n\in {\mathbb{N}}_{0}$, *b*, *c*, *d*, *e*, *f*, $q\in {\mathbb{C}}^{*}$, such that |q|≠1. Then, one has the following transformation formulas for a terminating _8_*W*_7_:

(32) $${\;}_{8}W_{7}\left(b;q^{-n},c,d,e,f;q,\frac{q^{n+2}b^{2}}{cdef}\right)$$

(33) $$=q^{{(}_{2}^{n})}{\left(\frac{-q^{2}b^{2}}{cdef}\right)}^{n}\frac{{\left(qb,b,c,d,e,f;q\right)}_{n}}{{\left(b;q\right)}_{2n}{\left(\frac{qb}{c},\frac{qb}{d},\frac{qb}{e},\frac{qb}{f};q\right)}_{n}}{\;}_{8}W_{7}\left(\frac{q^{-2n}}{b};q^{-n},\frac{q^{-n}c}{b},\frac{q^{-n}d}{b},\frac{q^{-n}e}{b},\frac{q^{-n}f}{b};q,\frac{q^{n+2}b^{2}}{cdef}\right)$$

(34) $$=\frac{{\left(\frac{qb}{ce},\frac{qb}{cf},qb,d,q\right)}_{n}}{{\left(\frac{qb}{c},\frac{qb}{e},\frac{qb}{f},\frac{d}{c};q\right)}_{n}}{\;}_{8}W_{7}\left(\frac{q^{-n}c}{d};q^{-n},\frac{q^{-n}c}{b},\frac{qb}{de},\frac{qb}{df},c;q,\frac{ef}{b}\right)$$

(35) $$=\frac{{\left(qb,\frac{q^{2}b^{2}}{cdef},q\right)}_{n}}{{\left(\frac{qb}{c},\frac{q^{2}b^{2}}{def},q\right)}_{n}}{\;}_{8}W_{7}\left(\frac{qb^{2}}{def};q^{-n},,\frac{qb}{de},\frac{qb}{df},\frac{qb}{ef},c;q,\frac{q^{n+1}b}{c}\right)$$

(36) $$=\frac{{\left(\frac{qb}{de},\frac{qb}{df},\frac{qb}{ef},qb;q\right)}_{n}}{{\left(\frac{qb}{def},\frac{qb}{d},\frac{qb}{e},\frac{qb}{f};q\right)}_{n}}{\;}_{8}W_{7}\left(\frac{q^{-n-1}def}{b};q^{-n},d,e,f,\frac{q^{-n-1}cdef}{b^{2}};q,\frac{q}{c}\right)$$

(37) =(qbcd,qb;q)n(qbc,qbd;q)nϕ4 3(q−n,qbef,c,dq−ncdb,qbe,qbf;q,q)

(38) =(qbef)n(qbcd,qb,e,f;q)n(qbc,qbd,qbe,qbf;q)nϕ4 3(q−n,q−ncb,q−ndb,qbefq−ncdb,q1−ne,q1−nf;q,q)

(39) =(q2b2cdef,qb,c;q)n(qbd,qbe,qbf;q)nϕ4 3(q−n,qbcd,qbce,qbcfq2b2cdef,q1−nc,qbc;q,q)

(40) =cn(qbcd,qbce,qbcf,qb;q)n(qbc,qbd,qbe,qbf;q)nϕ4 3(q−n,q−n−1cdefb2,q−ncb,cq−ncdb,q−nceb,q−ncfb;q,q).

**Proof.** Start with Theorem 3 and set $e^{2i\theta }=q^{n}b$, $a_{p}=q^{-\frac{n}{2}}\frac{c}{\sqrt{b}}$, $a_{r}=q^{-\frac{n}{2}}\frac{d}{\sqrt{b}}$, $a_{t}=q^{-\frac{n}{2}}\frac{e}{\sqrt{b}}$, $a_{u}=q^{-\frac{n}{2}}\frac{f}{\sqrt{b}}$Then, multiply every formula by the factor

$${\text{A}}_{n}\left(b,c,d,e,f|q\right):=\frac{q^{2{(}_{2}^{n})}{\left(-1\right)}^{n}{\left(qb\right)}^{\frac{5n}{2}}{\left(qb;q\right)}_{n}}{{\left(cdef\right)}^{n}{\left(\frac{qb}{c},\frac{qb}{d},\frac{qb}{e},\frac{qb}{f};q\right)}_{n}}.$$

With simplification, this completes the proof. □

**Remark 4.** Notice that in Corollary 3, our order of the representations begins with the principal _8_*W*_7_ representation in which the symmetry in the parameters c, *d*, *e*, *f* is evident and ends with the representation corresponding to the classical _4_*ϕ*_3_ basic hypergeometric representation of the Askey–Wilson polynomials (25). On the other hand, in Theorem 3, we have reversed the order of the corresponding representations. The reason why we have used the ordering as such is because the _4_*ϕ*_3_ representation of the Askey–Wilson polynomials (25) is historically first (see ([10] (1.8)) and the memoir ([11] (1.15))) and is certainly the most common (see e.g., ([1] (14.1.1))). The Askey–Wilson polynomials are symmetric in their four parameters, the _8_*W*_7_ representation in which this symmetry is evident demonstrates this symmetry. On the other hand, the polynomial nature of the Askey–Wilson polynomials is not clearly evident from the _8_*W*_7_ representation. In the first _4_*ϕ*_3_ representation, the polynomial nature of evident.

### 3.3. Terminating 4-Parameter Symmetric Interchange Transformations

The evidence that the first (and second) _8_*W*_7_ in Corollary 3 are symmetric in the variables *c*, *d*, *e*, *f* is clear. Therefore, all of the formulas in this corollary are invariant under the interchange of any two of those variables. This is true whether the symmetry between those variables is evident in the corresponding mathematical expression or not. Perhaps, the most famous parameter interchange transformation of this sort is Sears’ balanced _4_*ϕ*_3_ transformations ([8] (17.9.14)) which demonstrate the invariance (and provide specific transformation formulas) of the Askey–Wilson polynomials under parameter interchange. Other interesting parameter interchange transformations of this type can be obtained, such as by (34) with *c* ↔ *d* (preserves the argument), *c* ↔ *e*, *c* ↔ *f*, *d* ↔ *e*, *d* ↔ *f* interchanged (the invariance under the interchange *e* ↔ *f* is evident). Furthermore, when the symmetry within a set of variables is evident in the transformation corollaries presented below, then due to this symmetry, non-trivial transformation formulas can be obtained by equating the two expressions with certain variables interchanged.

In this subsection we present the entirety of all of the parameter interchange transformations for terminating basic hypergeometric transformations which arise from the Askey–Wilson polynomials.

**Corollary 4.** Let $n\in {\mathbb{N}}_{0}$, *b*, *c*, *d*, *e*, *f*, $q\in {\mathbb{C}}^{*}$, such that |q|≠1. Then, one has the following parameter interchange transformations for a terminating _8_*W*_7_:

(41) $${\;}_{8}W_{7}\left(\frac{q^{-n}c}{d},q^{-n},\frac{q^{-n}c}{b},\frac{qb}{de},\frac{qb}{df},c;q,\frac{ef}{b}\right)$$

(42) $$=\frac{{\left(\frac{qb}{de},\frac{qb}{df},\frac{qb}{c},\frac{d}{c},c;q\right)}_{n}}{{\left(\frac{qb}{ce},\frac{qb}{cf},\frac{qb}{d},\frac{c}{d},d;q\right)}_{n}}{\;}_{8}W_{7}\left(\frac{q^{-n}d}{c},q^{-n},\frac{q^{-n}d}{b},\frac{qb}{ce},\frac{qb}{cf},d;q,\frac{ef}{b}\right)$$

(43) $$=\frac{{\left(\frac{qb}{cd},\frac{qb}{e},\frac{d}{c},e;q\right)}_{n}}{{\left(\frac{qb}{ce},\frac{qb}{d},\frac{e}{c},d;q\right)}_{n}}{\;}_{8}W_{7}\left(\frac{q^{-n}c}{e},q^{-n},\frac{q^{-n}c}{b},\frac{qb}{ed},\frac{qb}{ef},c;q,\frac{df}{b}\right)$$

(44) $$=\frac{{\left(\frac{qb}{de},\frac{qb}{ef},\frac{qb}{c},\frac{e}{c},c;q\right)}_{n}}{{\left(\frac{qb}{cd},\frac{qb}{cf},\frac{qb}{e},\frac{c}{e},e;q\right)}_{n}}{\;}_{8}W_{7}\left(\frac{q^{-n}e}{c},q^{-n},\frac{q^{-n}e}{b},\frac{qb}{cd},\frac{qb}{cf},e;q,\frac{df}{b}\right)$$

(45) $$=\frac{{\left(\frac{qb}{ce},\frac{qb}{cd},\frac{qb}{f},\frac{c}{f},f;q\right)}_{n}}{{\left(\frac{qb}{ef},\frac{qb}{fd},\frac{qb}{c},\frac{c}{f},c;q\right)}_{n}}{\;}_{8}W_{7}\left(\frac{q^{-n}c}{f},q^{-n},\frac{q^{-n}c}{b},\frac{qb}{ef},\frac{qb}{df},c;q,\frac{de}{b}\right)$$

(46) $$=\frac{{\left(\frac{qb}{fd},\frac{qb}{fe},\frac{qb}{c},\frac{d}{c},c;q\right)}_{n}}{{\left(\frac{qb}{ce},\frac{qb}{cf},\frac{qb}{d},\frac{c}{f},d;q\right)}_{n}}{\;}_{8}W_{7}\left(\frac{q^{-n}f}{c},q^{-n},\frac{q^{-n}f}{b},\frac{qb}{cd},\frac{qb}{ce},f;q,\frac{de}{b}\right)$$

(47) $$=\frac{{\left(\frac{qb}{ef},\frac{d}{c};q\right)}_{n}}{{\left(\frac{qb}{cf},\frac{d}{e};q\right)}_{n}}{\;}_{8}W_{7}\left(\frac{q^{-n}e}{d},q^{-n},\frac{q^{-n}e}{b},\frac{qb}{dc},\frac{qb}{df},e;q,\frac{cf}{b}\right)$$

(48) $$=\frac{{\left(\frac{qb}{dc},\frac{qb}{df},\frac{qb}{e},\frac{d}{c},e;q\right)}_{n}}{{\left(\frac{qb}{ce},\frac{qb}{cf},\frac{qb}{d},\frac{e}{d},d;q\right)}_{n}}{\;}_{8}W_{7}\left(\frac{q^{-n}d}{e},q^{-n},\frac{q^{-n}d}{b},\frac{qb}{ec},\frac{qb}{ef},d;q,\frac{cf}{b}\right)$$

(49) $$=\frac{{\left(\frac{qb}{ef},\frac{d}{c};q\right)}_{n}}{{\left(\frac{qb}{ce},\frac{d}{f};q\right)}_{n}}{\;}_{8}W_{7}\left(\frac{q^{-n}f}{d},q^{-n},\frac{q^{-n}f}{b},\frac{qb}{dc},\frac{qb}{de},f;q,\frac{ce}{b}\right)$$

(50) $$=\frac{{\left(\frac{qb}{de},\frac{qb}{dc},\frac{qb}{f},\frac{d}{c},f;q\right)}_{n}}{{\left(\frac{qb}{ce},\frac{qb}{cf},\frac{qb}{d},\frac{f}{d},d;q\right)}_{n}}{\;}_{8}W_{7}\left(\frac{q^{-n}d}{f},q^{-n},\frac{q^{-n}d}{b},\frac{qb}{fc},\frac{qb}{fe},d;q,\frac{ce}{b}\right)$$

(51) $$=\frac{{\left(\frac{qb}{ed},\frac{qb}{f},\frac{d}{c},f;q\right)}_{n}}{{\left(\frac{qb}{cf},\frac{qb}{d},\frac{f}{d},d;q\right)}_{n}}{\;}_{8}W_{7}\left(\frac{q^{-n}e}{f},q^{-n},\frac{q^{-n}e}{b},\frac{qb}{fc},\frac{qb}{fd},e;q,\frac{cd}{b}\right)$$

(52) $$=\frac{{\left(\frac{qb}{df},\frac{qb}{e},\frac{d}{c},e;q\right)}_{n}}{{\left(\frac{qb}{ce},\frac{qb}{d},\frac{e}{d},d;q\right)}_{n}}{\;}_{8}W_{7}\left(\frac{q^{-n}f}{e},q^{-n},\frac{q^{-n}e}{b},\frac{qb}{ec},\frac{qb}{ed},f;q,\frac{cd}{b}\right).$$

**Proof.** Start with (34) and consider all permutations of the symmetric parameters *c*, *d*, *e*, *f* which produce non-trivial transformations. The ordering of the elements is given by the first argument of the _8_*W*_7_ as follows: {(*c*, *d*), (*d*, *c*), (*c*, *e*), (*e*, *c*), (*c*, *f*), (*f*, *c*), …, (*e*, *f*), (*f*, *e*)}. □

**Corollary 5.** Let $n\in {\mathbb{N}}_{0}$, *b*, *c*, *d*, *e*, *f*, q∈ℂ*, such that |q|≠1. Then, one has the following parameter interchange transformations for a terminating _8_*W*_7_:

(53) $${\;}_{8}W_{7}\left(\frac{qb^{2}}{def};q^{-n},\frac{qb}{de},\frac{qb}{df},\frac{qb}{ef},c;q,\frac{q^{n+1}b}{c}\right)$$

(54) $$=\frac{{\left(\frac{qb}{c},\frac{q^{2}b^{2}}{def};q\right)}_{n}}{{\left(\frac{qb}{d},\frac{q^{2}b^{2}}{cef};q\right)}_{n}}{\;}_{8}W_{7}\left(\frac{qb^{2}}{cef};q^{-n},\frac{qb}{ce},\frac{qb}{cf},\frac{qb}{ef},d;q,\frac{q^{n+1}b}{d}\right)$$

(55) $$=\frac{{\left(\frac{qb}{c},\frac{q^{2}b^{2}}{def};q\right)}_{n}}{{\left(\frac{qb}{e},\frac{q^{2}b^{2}}{cdf};q\right)}_{n}}{\;}_{8}W_{7}\left(\frac{qb^{2}}{cdf};q^{-n},\frac{qb}{cd},\frac{qb}{cf},\frac{qb}{df},e;q,\frac{q^{n+1}b}{e}\right)$$

(56) $$=\frac{{\left(\frac{qb}{c},\frac{q^{2}b^{2}}{def};q\right)}_{n}}{{\left(\frac{qb}{f},\frac{q^{2}b^{2}}{cde};q\right)}_{n}}{\;}_{8}W_{7}\left(\frac{qb^{2}}{cde};q^{-n},\frac{qb}{cd},\frac{qb}{ce},\frac{qb}{de},f;q,\frac{q^{n+1}b}{e}\right).$$

**Proof.** Start with (35) and consider all permutations of the symmetric parameters *c*, *d*, *e*, *f* which produce non-trivial transformations. □

**Remark 5.** Another set of parameter interchange transformations can be obtained by considering all permutations of the symmetric parameters *c*, *d*, *e*, *f* in (36). However, one can see that these are equivalent to the above Corollary 5 by replacing

$$\left(b,c,d,e,f\right)\mapsto \left(\frac{q^{-1-2n}def}{b^{2}},\frac{q^{-1-n}cdef}{b^{2}},\frac{q^{-n}f}{b},\frac{q^{-n}e}{b},\frac{q^{-n}d}{b}\right).$$

**Corollary 6.** Let $n\in {\mathbb{N}}_{0}$, *b*, *c*, *d*, *e*, *f*, $q\in {\mathbb{C}}^{*}$, such that |q|≠1. Then, one has the following parameter interchange transformations for a terminating _4_*ϕ*_3_:

(57) ϕ4 3(q−n,qbef,c,dq−ncdb,qbe,qbf;q,q)

(58) =(qbde,qbc;q)n(qbcd,qbe;q)nϕ4 3(q−n,qbcf,d,eq−ndeb,qbc,qbf;q,q)

(59) =(qbdf,qbc;q)n(qbcd,qbf;q)nϕ4 3(q−n,qbce,d,fq−ndfb,qbc,qbe;q,q)

(60) =(qbce,qbb;q)n(qbcd,qbe;q)nϕ4 3(q−n,qbdf,c,eq−nceb,qbd,qbf;q,q)

(61) =(qbcf,qbd;q)n(qbcd,qbf;q)nϕ4 3(q−n,qbde,c,fq−ncfb,qbd,qbe;q,q)

(62) =(qbef,qbc,qbd;q)n(qbde,qbe,qbf;q)nϕ4 3(q−n,qbcd,e,fq−nefb,qbc,qbd;q,q).

**Proof.** Start with (37) and consider all permutations of the symmetric parameters *c*, *d*, *e*, *f* which produce non-trivial transformations. □

**Remark 6.** Another set of parameter interchange transformations can be obtained by considering all permutations of the symmetric parameters *c*, *d*, *e*, *f* in (38). However, one can see that these are equivalent to the above Corollary 6 by replacing

$$\left(b,c,d,e,f\right)\mapsto \left(\frac{q^{-2n}}{b},\frac{q^{-n}c}{b},\frac{q^{-n}d}{b},\frac{q^{-n}e}{b},\frac{q^{-n}f}{b}\right).$$

**Corollary 7.** Let $n\in {\mathbb{N}}_{0}$, *b*, *c*, *d*, *e*, *f*, $q\in {\mathbb{C}}^{*}$, such that |q|≠1. Then, one has the following parameter interchange transformations for a terminating _4_*ϕ*_3_:

(63) ϕ4 3(q−n,qbcd,qbce,qbcfq2b2cdef,q1−nc,qbc;q,q)

(64) =(qbd,d;q)n(qbc,c;q)nϕ4 3(q−n,qbdc,qbde,qbdfq2b2cdef,q1−nd,qbd;q,q)

(65) =(qbe,e;q)n(qbc,c;q)nϕ4 3(q−n,qbec,qbed,qbefq2b2cdef,q1−ne,qbe;q,q)

(66) =(qbf,f;q)n(qbc,c;q)nϕ4 3(q−n,qbfc,qbfd,qbfeq2b2cdef,q1−nf,qbf;q,q).

**Proof.** Start with (39) and consider all permutations of the symmetric parameters *c*, *d*, *e*, *f* which produce non-trivial transformations. □

**Remark 7.** Another set of parameter interchange transformations can be obtained by considering all permutations of the symmetric parameters *c*, *d*, *e*, *f* in (40). However, one can see that these are equivalent to the above Corollary 7 by replacing

$$\left(b,c,d,e,f\right)\mapsto \left(\frac{q^{-n}f}{e},\frac{qb}{ce},\frac{qb}{de},f,\frac{q^{-n}f}{b}\right).$$

The *q*-inverse Askey–Wilson polynomials are simply a scaled version of the Askey–Wilson polynomials with the free parameters *a*_*k*_ replaced by their reciprocals $a_{k}^{-1}$. We demonstrate this in the following remark.

**Remark 8.** Let *p*_*n*_(*θ*; *a*_1_, *a*_2_, *a*_3_, *a*_4_|*q*) := *p*_*n*_(*x*; **a**|*q*), where *x* = cos *θ*, be any representation of the Askey–Wilson polynomials. Then the q-inverse Askey–Wilson polynomials *p*_*n*_(*x*; **a**|*q*^−1^) are given by

$$\begin{array}{l} p_{n}\left(\theta ;a_{1},a_{2},a_{3},a_{4}|q\right):=q^{-3{(}_{2}^{n})}{\left(-a_{1234}\right)}^{n}p_{n}\left(-\theta ;a_{1}^{-1},a_{2}^{-1},a_{3}^{-1},a_{4}^{-1}|q\right)\\ \;\;\;\;\;\;=q^{-3{(}_{2}^{n})}{\left(-a_{1234}\right)}^{n}p_{n}\left(\theta ;a_{1}^{-1},a_{2}^{-1},a_{3}^{-1},a_{4}^{-1}|q\right), \end{array}$$

where the second equality follows from Remark 1. So aside from a specific normalization, as is well-known, the q-inverse Askey–Wilson polynomials are the Askey–Wilson polynomials with the parameters taken to be their reciprocals. Note that this will not be the case for the symmetric subfamilies of the Askey–Wilson polynomials.

Nonetheless, we give in the following corollary the terminating basic hypergeometric representations of these polynomials.

**Corollary 8.** Let *p*_*n*_(*x*, **a**|*q*) and all the respective parameters be defined as previously. Then, the q-inverse Askey–Wilson polynomials are given by:

(67) pn(x;a|q−1)=q−3(2n)(−apa1234)n({1aps}s≠p;q)nϕ4 3(q−n,qn−1a1234,e±iθap{1aps}s≠p;q,q)

(68) =q−4(2n)(apa1234)n(qn−1a1234,e±iθap;q)nϕ4 3(q−n,{qn−1aps}s≠pq2−2na1234,q1−nape±iθ;q,q)

(69) =q−3(2n)(−a1234e−iθ)n(1apr,eiθat,eiθau;q)nϕ4 3(q−n,e−iθap,e−iθar,q1−natu1apr,q1−nate−iθ,q1−naue−iθ;q,q)

(70) =q−3(2n)(−a1234e−iθ)n(1qa1234;q)2n({eiθas}s≠p,apeiθqa1234;q)n(1qa1234;q)n(apeiθqa1234;q)2n    ×W87(q1−2na1234e−iθap;q−n,{q1−na1234aps}s≠p,e−iθap;q,qape−iθ)

(71) $$=q^{-3{(}_{2}^{n})}{\left(-a_{1234}e^{-i\theta }\right)}^{n}\frac{{\left(\frac{e^{i\theta }}{a_{p}},{\left\{\frac{a_{ps}}{a_{1234}}\right\}}_{s\neq p};q\right)}_{n}}{{\left(\frac{a_{p}e^{-i\theta }}{a_{1234}};q\right)}_{n}}\;\;\;\;\;\times {\;}_{8}W_{7}\left(\frac{a_{p}e^{-i\theta }}{qa_{1234}};q^{-n},{\left\{\frac{e^{-i\theta }}{a_{s}}\right\}}_{s\neq p},\frac{q^{n-1}}{a_{1234}};q,qa_{p}e^{i\theta }\right)$$

(72) $$=q^{-3{(}_{2}^{n})}{\left(-a_{p}a_{1234}\right)}^{n}\frac{{\left(\frac{1}{a_{pt}},\frac{1}{a_{pu}},\frac{e^{\pm i\theta }}{a_{r}};q\right)}_{n}}{{\left(\frac{a_{p}}{a_{r}};q\right)}_{n}}\;\;\;\;\;\times {\;}_{8}W_{7}\left(\frac{q^{-n}a_{r}}{a_{p}};q^{-n},q^{1-n}a_{rt},q^{1-n}a_{ru},\frac{e^{\pm i\theta }}{a_{p}};q,\frac{q^{n}}{a_{tu}}\right)$$

(73) $$=q^{-3{(}_{2}^{n})}{\left(-a_{1234}e^{-i\theta }\right)}^{n}\frac{{\left(\left\{\frac{e^{i\theta }}{a_{s}}\right\};q\right)}_{n}}{{\left(e^{2i\theta };q\right)}_{n}}{\;}_{8}W_{7}\left(q^{-n}e^{-2i\theta };q^{-n},\left\{\frac{e^{-i\theta }}{a_{s}}\right\};q,q^{2-n}a_{1234}\right).$$

**Proof.** Applying Theorem 2 to the terminating basic hypergeometric representations of the Askey–Wilson polynomials (25)–(31) produces the inverted basic hypergeometric representations (67)–(73). This completes the proof. □
